# Melatonin Improves Outcomes of Heatstroke in Mice by Reducing Brain Inflammation and Oxidative Damage and Multiple Organ Dysfunction

**DOI:** 10.1155/2013/349280

**Published:** 2013-12-04

**Authors:** Yu-Feng Tian, Cheng-Hsien Lin, Shu-Fen Hsu, Mao-Tsun Lin

**Affiliations:** ^1^Division of General Surgery, Department of Surgery, Chi Mei Medical Center, Tainan 710, Taiwan; ^2^Department of Health and Nutrition, Chia Nan University of Pharmacy and Science, Tainan 717, Taiwan; ^3^Department of Nursing, Shu-Zen Junior College of Medicine and Management, Kaohsiung 821, Taiwan; ^4^Department of Medical Research, Chi Mei Medical Center, Tainan 710, Taiwan

## Abstract

We report here that when untreated mice underwent heat stress, they displayed thermoregulatory deficit (e.g., animals display hypothermia during room temperature exposure), brain (or hypothalamic) inflammation, ischemia, oxidative damage, hypothalamic-pituitary-adrenal axis impairment (e.g., decreased plasma levels of both adrenocorticotrophic hormone and corticosterone during heat stress), multiple organ dysfunction or failure, and lethality. Melatonin therapy significantly reduced the thermoregulatory deficit, brain inflammation, ischemia, oxidative damage, hypothalamic-pituitary-adrenal axis impairment, multiple organ dysfunction, and lethality caused by heat stroke. Our data indicate that melatonin may improve outcomes of heat stroke by reducing brain inflammation, oxidative damage, and multiple organ dysfunction.

## 1. Introduction

Melatonin, the main product of the pineal gland, is found in high concentrations in other body fluids and tissues [1, 2] and possesses anti-inflammatory and antioxidant actions [3–6]. We have evaluated the prophylactic [7] as well as the therapeutic [8] effect of melatonin in heatstroke rats under general anesthesia and showed the therapeutic effects of melatonin on heatstroke-induced multiple organ dysfunction syndrome. According to a more recent review [9], the ischemic and oxidative damage to the hypothalamus during heatstroke may cause multiple organ dysfunction or failure through hypothalamic-pituitary-adrenal (HPA) axis mechanisms. Studies are warranted to know whether the heatstroke-induced brain (or hypothalamic) inflammation and damage, thermoregulatory deficits, and multiple organ dysfunction can be affected by melatonin therapy in an unanesthetized and unrestrained mouse model [10–12].

To deal with the hypothesis, we assessed the temporal profiles of cellular markers of ischemia (e.g., glutamate and lactate/pyruvate ratio), damage (e.g., glycerol), inflammation (e.g., tumor necrosis factor-alpha (TNF-*α*), interleukin-1 (IL-1*β*), IL-10, and myeloperoxidase (MPO) activity), and oxidative damage (e.g., prooxidant enzymes (e.g., lipid peroxidation and glutathione oxidation), anti-oxidant defenses (e.g., glutathione peroxidase (GP_*x*_), and glutathione reductase (GR), oxidant radicals, nitric oxide metabolites (NO_*x*_), and dihydroxybenzoic acid (DHBA)) in the hypothalamus that occurred after heat regimen in mice treated with or without melatonin therapy. In addition, the influence of melatonin therapy on the heatstroke-induced thermoregulatory deficits as well as increased plasma levels of multiple organ dysfunction or failure [10–12] was assessed.

## 2. Materials and Methods

### 2.1. Mice

Present studies were performed in male ICR mice (29–37 g), whose stock originated from the Institute of Cancer Research of the National Institutes of Health in the USA. They were purchased from the National Animal Center (Taipei, Taiwan) and kept under a 12-hour light-dark cycle at controlled temperature (21 ± 2°C) with free access to food and tap water.

### 2.2. Murine Model of Heatstroke

Institute of Cancer Research male mice 8 to 10 weeks old were exposed to whole body heating (WBH; 41.2°C; relative humidity 50%–55%; 1 h) in an environment-controlled chamber [10–12]. The heat-stressed mice were returned to the normal room temperature (26°C) after the end of the heat exposure. Mice that survived to day 4 of heat treatment were considered survivors, and the data were used for analysis of the results. Core temperatures were measured every 1 min with a copper constant thermocouple inserted into the rectum and connected to a thermometer (HR1300; Yokogawa, Tokyo, Japan). Before the start of thermal experiments, mice were housed at an ambient temperature (26°C) below the neutral zone for this species. After 1-hour heating period, animals were properly fed and hydrated. In separate experiments, 4 h post-WBH, animals were sacrificed and their brains and blood were obtained for biochemical verification [10–12].

### 2.3. Experimental Groups

Three hundred mice were randomly divided into 3 major groups: (a) nonheated mice treated with vehicle solution  (*n* = 60): these groups of animals were s.c. injected with one dose of 0.2 mL of 0.25% ethanol-saline [13] immediately post-WBH; (b) heated mice treated with vehicle solution  (*n* = 60); and (c) heated mice treated with melatonin (N-acetyl-5-methoxytryptamine) (0.2 mg/kg, 1 mg/kg, or 5 mg/kg) [14] immediately post-WBH  (*n* = 180).

In Experiment  1, effects of heat exposure on body core temperature and % survival of different groups of mice were assessed  (*n* = 60).

In Experiment  2, effects of heat exposure on cellular ischemia and damage markers in brain (or hypothalamus) of different groups of mice were measured  (*n* = 60).

In Experiment  3, effects of heat exposure on inflammatory mediators in brain (or hypothalamus) of different groups of mice were measured  (*n* = 60).

In Experiment  4, effects of heat exposure on oxidative stress markers in brain (or hypothalamus) of different groups of mice were measured  (*n* = 60).

In Experiment  5, effects of heat exposure on serum levels of multiple organ dysfunction indicators, ACTH, and corticosterone of different groups were measured  (*n* = 60).

### 2.4. Extracellular Levels of Glutamate, Lactate-to-Pyruvate Ratio, Glycerol, Nitric Oxide, and Hydroxyl Radicals in the Hypothalamus

Hypothalamic samples were homogenized in 0.05 M phosphate buffer at pH7.0 and then centrifuged at 4000 ×g for 20 min at 4°C. The supernatants were used for the determination of cellular levels of glutamate, lactate-to-pyruvate ratio, glycerol, nitric oxide, and hydroxyl radicals. The dialysis probe (4 nm in length c CMA/12; Carnegie Medicine, Stockholm, Sweden) was put into the supernatants to obtain the dialysates.

The nitric oxide (NO_*x*_^−^) concentration in the dialysates of hypothalamus was measured with the Eicom ENO-20  NO_*x*_^−^  analysis system (Eicom, Kyoto) [15]. In the Eicom ENO-20  NO_*x*_^−^  analysis system, after the  NO_2_^−^  and  NO_3_^−^  in the sample have been separated by the column, the  NO_2_^−^  reacts in the acidic solution with the primary aromatic amine to produce an azo compound. Following this, the addition of aromatic amines to the azo compound results in a coupling that produces a diazo compound, and the absorbance rate of the red color in this compound is then measured. For measurement of glutamate, lactate-to-pyruvate ratio, and glycerol, the dialysates were injected onto a CMA600 microdialysis analyzer (Carnegie Medicine, Stockholm, Sweden). The concentrations of hydroxyl radicals were measured by a modified procedure based on the hydroxylation of sodium salicylates by hydroxyl radicals, leading to the production of 2,3-dihydroxybenzoic acid and 2,5-dihydroxybenzoic acid [16].

### 2.5. Determination of Lipid Peroxidation

Lipid peroxidation was assessed by measuring the levels of malondialdehyde (MDA) with 2-thiobarbituric acid (TBA) to form a chromophore absorbing at 532 nm [17]. About 0.1 g of tissue was homogenized with 1.5 mL of 0.1 M phosphate buffer at pH3.5. The reaction mixture (0.2 mL of sample, 1.5 mL of 20% acetic acid, 0.2 mL of 8.1% sodium dodecyl sulfate, and 1.5 mL of aqueous solution of 0.8% TBA, up to 4 mL with distilled water) was heated to 95°C for 1 h, and then 5 mL of N-butanol and pyridine (15 : 1 vol/vol) was added. The mixture was vortexed vigorously and centrifuged at 1500 g for 10 min, and the absorbance of the organic phase was measured at 532 nm. The values were expressed as nanomoles of TBA-reactive substances (MDA equivalent) per milligram of protein.

### 2.6. Quantification of Total and Oxidized Glutathione

Tissues were homogenized in 5% 5-sulfosalicylic acid (1 : 10 wt/vol) at 0°C, and the supernatants were used for analysis of total and oxidized glutathione. Total glutathione (reduced-form glutathione (GSH) + oxidized-form glutathione (GSSG)) was analyzed according to the Tietze method [18], and GSSG was determined as described by Griffith [19]. The recycling assay for total glutathione is oxidized by 5,5-Dithiobis (2 acid) (DTNB) to give GSSG with stoichiometric formation of 5-thio-2-nitrobenzoic acid. GSSG is reduced to GSH by the action of the highly specific glutathione reductase (GR) and nicotinamide adenine dinucleotide phosphate (reduced form; NADPH). The rate of 5-thio-2-nitrobenzoic acid formation is followed at 412 nm and is proportional to the sum of GSH and GSSG present.

### 2.7. Determination of Glutathione Peroxidase (GP_*x*_) and Glutathione Reductase (GR) Activity

Tissues were homogenized in 0.05 M phosphate buffer, pH7.0 and then centrifuged at 4000 ×g for 20 min at 4°C. The supernatants were used for GP_*x*_ and GR activity assay. The GP_*x*_ and GR activities were performed with a commercial GP_*x*_ assay kit (Sigma, USA) and a GP assay kit (Sigma, USA), respectively. One unit of GP_*x*_ and GR activity was defined as the amount of sample required to oxidize 1 mmol of NADPH per minute based on the molecular absorbance of 6.22 × 10^6^ for NADPH.

### 2.8. Myeloperoxidase Activity

MPO activity, an indicator of polymorphonuclear leukocyte accumulation, was determined in the hypothalamus as described previously [20] at 4 hours after heat stress. MPO activity was defined as the quantity of enzyme degrading 1 *μ*mol of peroxide/min at 37°C and was expressed in milliunits/gram of wet tissue.

### 2.9. Determination of Cytokines in the Hypothalamus

The hypothalamic samples were prepared according to previous reports [21]. The tissues were homogenized in five volumes of ice-cold Ripa buffer. The homogenates were incubated on ice for 30 min and then centrifuged (15,000 ×g, 30 min, 4°C) twice. The concentrations of these cytokines in the supernatants were determined by commercially available ELISA kits (R & D Systems, Minneapolis, MN, USA) according to the manufacturer's instructions. Optical densities were read on a plate reader set at 450 nm for these cytokines. The concentrations of these cytokines in the samples were calculated from the standard curve multiplied by the dilution factor and were expressed as pg/g.

### 2.10. Quantification of Multiple Organ Dysfunction and Injury

Creatinine, blood urea nitrogen (BUN), alanine aminotransferase (ALT), aspartate aminotransferase (AST), and alkaline phosphatase (ALP) were estimated in blood samples collected 4 hours after the start of heat stress or the equivalent time point for the nonheated animals. The serum levels of creatinine, BUN, ALT, AST, and ALP were determined by spectrophotometry (HITACHI 7600, Tokyo, Japan).

### 2.11. Plasma Assessment of Corticosterone and Adrenocorticotrophic Hormone (ACTH)

Plasma corticosterone and ACTH were assessed using corticosterone Double Antibody RIA kit (MP Biomedicals, Solon, Oh, USA) and ACTH (Rat, Mouse)-RIA kit (Phoenix Pharmaceuticals, Burlingame, CA, USA), respectively. All analyses were performed according to manufacturer's instructions.

### 2.12. Statistical Analysis

All values in the tables and text are expressed as mean ± S.E.M. of *n* observations, where *n* represents the number of animals studied. Statistical evaluation was performed by using analysis of variance (ANOVA) followed by a multiple-comparison test (Scheffe's test). A  *P*  value of less than 0.05 was considered to be statistically significant.

## 3. Results

### 3.1. Melatonin Prevents Heat-Induced Hypothermia and Lethality

**Figure 1:**
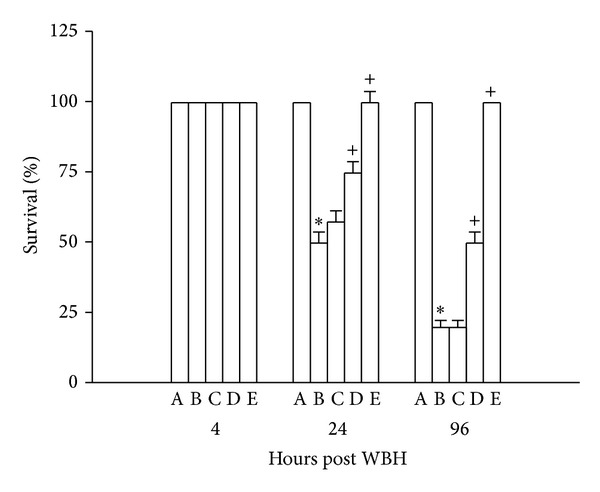
Percent survival values for nonheated mice untreated (A), heated mice treated with vehicle solution (B), heated mice treated with melatonin (0.2 mg/kg) (C), heated mice treated with melatonin (1 mg/kg) (D), and heated mice treated with melatonin (5 mg/kg) (E) 4–96 hours after whole body heating (WBH). Data are expressed as means ± S.E.M. for 12 mice per group. *P < 0.05  compared with nonheated mice untreated mice; ^+^ *P* < 0.05  compared with heated mice treated with vehicle solution.

As summarized in Table 1, the body core temperature values of heated mice were significantly lower than those of nonheated mice kept at a normal ambient temperature (26°C) (33.2 ± 0.2°C and 37.2 ± 0.3°C, resp.) (*P* < 0.001). Additionally, the fraction survival of heated mice was significantly lower than those of nonheated mice (1/12 and 12/12, resp.) (*P* < 0.001) (Figure 1). The heat-induced hypothermia and lethality were significantly reduced by melatonin therapy (Table 1 and Figure 1).

### 3.2. Melatonin Reduces Heat-Induced Increased Levels of Glutamate, Lactate-to-Pyruvate Ratio, Glycerol, Nitrite (NO), and Dihydroxybenzoic Acid (DHBAs) in the Hypothalamus

Table 2 summarizes the effects of heat exposure on cellular levels of glutamate, lactate-to-pyruvate ratio, glycerol, nitrite, and DHBA in the hypothalamus in different groups of mice. As compared with nonheated mice, the heated mice had higher levels of glutamate, lactate-to-pyruvate ratio, glycerol, nitrite, and DHBA in the hypothalamus (*P* < 0.05). The heat-induced increased levels of glutamate, lactate-to-pyruvate ratio, glycerol, nitrite, and DHBA in the hypothalamus were all significantly and dose-dependently reduced by melatonin (0.2–5.0 mg/kg).

### 3.3. Melatonin Attenuates Heat-Induced Increased Levels of TNF-*α*, IL-1*β*, and MPO but Increases Production of IL-10 in the Hypothalamus

As shown in Table 3, hypothalamic levels of TNF-*α*, IL-1*β*, and MPO were all increased in heated mice. Again, heat-induced overproduction of TNF-*α*, IL-1*β*, and MPO was significantly and dose-dependently reduced by melatonin therapy (0.2–5.0 mg/kg) (*P* < 0.05). In contrast, hypothalamic levels of IL-10 were significantly and dose-dependently increased by melatonin treatment in heated mice.

### 3.4. Melatonin Decreases Heat-Induced Hypothalamic Oxidative Stress

As shown in Table 4, the hypothalamic levels of both MDA and GSSG/GSH in heated mice were significantly higher than those of nonheated mice. In contrast, the hypothalamic levels of GP, GR of heated mice were significantly higher than those of nonheated mice (*P* < 0.05). The heat-induced increased levels of MDA and GSSG/GSH as well as the decreased levels of GP, GR in the hypothalamus were significantly and dose-dependently reduced by melatonin therapy (0.2–5.0 mg/kg) (*P* < 0.05).

### 3.5. Melatonin Attenuates Heat-Induced Increased Plasma Levels of Multiple Organ Injury Markers

The plasma levels of BUN, creatinine, ALT, AST, and AP in heated mice were all significantly higher than those of nonheated mice (Table 5). The heat-induced increased plasma levels of these parameters were all significantly and dose-dependently reduced by melatonin treatment (0.2–5.0 mg/kg) (*P* < 0.05).

### 3.6. Melatonin Enhances Heat-Induced Increased Plasma Levels of Both ACTH and Corticosterone

As shown in Table 6, the plasma levels of both ACTH and corticosterone in heated mice were significantly higher than those of nonheated controls (*P* < 0.05). The heat-induced increased plasma levels of both ACTH and corticosterone were all significantly enhanced by melatonin therapy (*P* < 0.05; Table 6).

## 4. Discussion

According to Chatterjee et al. [10, 11], heat-treated mice display body core temperatures of >40°C immediately after the termination of WBH and profound hypothermia at +4, +6, and +20 h after. This is confirmed by the present results. We further demonstrate that heat-induced hypothermia in mice can be significantly and dose-dependently prevented by melatonin therapy. However, the contention is not consistent with the findings of Leon et al. [22], who reported on mice exposed to an ambient temperature of ~39.5°C until a maximal core temperature of 42.7°C was attained. During their recovery, the mice had hypothermia (29.3°C) and, after 24 h of recovery, a fever-like elevation (37.8°C).

The hypothalamus is believed to be involved in regulating homeostasis, motivation, and emotional behavior; these functions are mediated through hypothalamic control of autonomic and endocrine activity [23]. The hypothalamus allows the output of pituitary hormones to response to changes in the autonomic nervous system activity and to the needs of temperature regulation, water balance, and energy requirements. Heat exposure is a stimulus that triggers biological stress reactions [24]. The hypothalamo-pituitary-adrenocortical (HPA) axis is also mobilized, as suggested by the increase in c-fos-positive cells [25] and c-fos mRNA content [26] in the hypothalamic paraventricular nucleus, as well as the increase in blood adrenocorticotrophic-hormone (ACTH) and corticosterone concentrations [27, 28]. Decreased heat tolerance has been associated with HPA axis impairment [29]. More than half a century ago, thermal injury to the thermoregulatory centers of the hypothalamus was hypothesized to be the primary mechanism of mortality [30]. Indeed, according to a more recent review [9], ischemic and oxidative damage to the hypothalamus may be responsible for heatstroke. Severe heat stress increases cutaneous blood flow and metabolism and decreases splanchnic blood flow. Severe heat stress also decreases mean arterial pressure, increases intracranial pressure, and decreases cerebral perfusion pressure, all of which lead to cerebral ischemia and hypoxia. Compared with normothermic controls, rodents with heatstroke have higher values of cellular ischemia (e.g., glutamate and lactate-to-pyruvate ratio) and damage (e.g., glycerol) markers, prooxidant enzymes (e.g., lipid peroxidation and glutathione oxidation markers), proinflammatory cytokines (e.g., interleukin-1*β* and tumor necrosis factor-*α*), inducible nitric oxide-synthase-dependent nitric oxide and an indicator for the accumulation of polymorphonuclear leukocytes (e.g., myeloperoxidase activity) as well as neuronal damage (e.g., apoptosis and necrosis) in the hypothalamus after heat stroke. Hypothalamic values of antioxidant defenses (e.g., glutathione peroxidase and glutathione reductase), however, are lower. Melatonin therapy, in addition to attenuating heat-induced hypothermia (thermoregulatory deficits), significantly attenuates heat-induced inflammatory, ischemic and oxidative damage to the hypothalamus.

Furthermore, our present results demonstrated that melatonin therapy significantly enhanced HPA axis mechanisms (as reflected by increased plasma levels of both ACTH and corticosterone in response to heat stress), led to reduction of multiple organ dysfunction or failure (as reflected by decreased plasma levels of BUN, creatinine, ALT, AST, and ALP), and resulted in attenuation of lethality in heatstroke mice.

Our present results are consistent with many previous investigations. For example, melatonin improved the clinical outcome of the septic newborns as judged by measurement of sepsis-related serum parameters via reducing the serum levels of lipid peroxidation products [31]. A major brain metabolite of melatonin acts as a potent nitric oxide scavenger, inhibitor, and/or downregulator of neuronal and inducible nitric oxide synthase and as a mitochondrial metabolism modulator [32]. Melatonin protected against mitochondrial reactive oxygen species-mediated apoptosis in astrocytes [33] and isoproterenol-induced myocardial injury in the rat via its antioxidative mechanism [34]. In addition, melatonin protected the mitochondria from oxidative damage by preventing cardiolipin [35]. Melatonin also may attenuate peritonitis-induced lethality in conscious rats by exerting its antioxidant effect [36]. These observations prompted us to think that these multiple mitochondrial layers of protection provided by melatonin may be crucial for future therapeutic prevention and treatment of heatstroke.

Systemic inflammatory response syndrome is characterized by increased serum levels of TNF-*α*, ICAM-1, E-selectin, IL-1*β*, and IL-6 [37–40]. Increased levels of these systemic inflammatory response syndrome molecules have also been shown in patients [41] or rats [42]. In addition, the increased serum levels of these molecules during heatstroke in an anesthetized rat model could be reduced by melatonin treatment [7]. The present results further showed that the increased levels of some of these molecules in the hypothalamus in a mice could also be reduced by melatonin. Additionally, melatonin therapy increased the hypothalamic levels of IL-10 in heatstroke, which was believed to be anti-inflammatory cytokine [43]. These results indicate that melatonin may reduce heat-induced activated inflammation by reducing the levels of these systemic inflammatory response molecules in the hypothalamus. It is known that melatonin intensifies the expression of protective heat shock proteins [44] and that it enhances heat shock protein 27 expression [45] as well as having anti-inflammatory properties and antioxidant properties.

## 5. Conclusion

In summary, we report here that when untreated mice underwent heat stress, they displayed thermoregulatory deficit (e.g., animals display hypothermia during room temperature exposure), brain (or hypothalamic) inflammation, ischemia, and oxidative damage, HPA axis impairment, multiple organ dysfunction or failure, and lethality. Melatonin therapy may improve outcomes of heatstroke in mice by reducing brain inflammation and oxidative damage, and multiple organ dysfunction.

## Figures and Tables

**Table 1:** Effects of heat exposure on body core temperatures of different groups of mice.

Treatment groups	Core temperature (°C)	*P* values
(1) Nonheated mice untreated	37.2 ± 0.3	
(2) Heated mice treated with vehicle saline	33.2 ± 0.2	^a^ *P* < 0.01
(3) Heated mice treated with melatonin 0.2 mg/kg	33.5 ± 0.3	^a^ *P* < 0.01
(4) Heated mice treated with melatonin 1 mg/kg	36.3 ± 0.3	^b^ *P* < 0.01
(5) Heated mice treated with melatonin 5 mg/kg	37.4 ± 0.4	^b^ *P* < 0.01

Core temperatures were measured 4 hours after whole body heating for heated groups or the equivalent time period for non-heated groups. ^a^Compared with non-heated groups; ^b^Compared with group 2. Data are means ± S.E.M. of 12 mice per group.

**Table 2:** Effect of heat exposure on hypothalamic levels of various parameters in different groups of mice.

Treatment groups	Glutamate (% of baseline)	Lactate/pyruvate ratio	Glycerol (% of baseline)	Nitric oxide (**µ**M)	2,3-DHBA (% of baseline)
(1) Non-heated mice	100 ± 6	10 ± 3	100 ± 5	16 ± 2	100 ± 5
(2) Heated mice treated with vehicle solution	207 ± 11^a^	219 ± 15^a^	173 ± 7^a^	99 ± 11^a^	151 ± 8^a^
(3) Heated mice treated with melatonin 0.2 mg/kg	214 ± 12	206 ± 14	214 ± 11	106 ± 10	165 ± 7
(4) Heated mice treated with melatonin 1 mg/kg	141 ± 8^b^	87 ± 5^b^	135 ± 5^b^	61 ± 4^b^	118 ± 5^b^
(5) Heated mice treated with melatonin 5 mg/kg	106 ± 5^b^	10 ± 2^b^	102 ± 4^b^	15 ± 2^b^	99 ± 6^b^

Samples were measured 4 hours after whole body heating or the equivalent time period for non-heated groups. ^a^Compared with non-heated groups (*P* < 0.05); ^b^Compared with group 2 (*P* < 0.05). Data are means ± S.E.M. of 12 mice per group.

**Table 3:** Effects of heat exposure on hypothalamic levels of various parameters in different groups of mice.

Treatment groups	TNF-*α* (pg/g)	IL-1*β* (pg/g)	IL-10 (pg/g)	MPO activity (pg/wet tissue)
(1) Non-heated mice	10 ± 3	8 ± 2	5 ± 2	51 ± 8
(2) Heated mice treated with vehicle solution	208 ± 18^a^	426 ± 51^a^	6 ± 3	448 ± 14
(3) Heated mice treated with melatonin 0.2 mg/kg	224 ± 21	453 ± 56	4 ± 2	507 ± 17
(4) Heated mice treated with melatonin 1 mg/kg	88 ± 8^b^	190 ± 18^b^	212 ± 22^b^	144 ± 9^b^
(5) Heated mice treated with melatonin 5 mg/kg	9 ± 3^b^	8 ± 3^b^	256 ± 30^b^	51 ± 11^b^

Samples were measured 4 hours after whole body heating for non-heated groups. ^a^Compared with non-heated groups (*P* < 0.05); ^b^Compared with group 2 (*P* < 0.05). Data are means ± S.E.M. of 12 mice per group.

**Table 4:** Effects of heat exposure on hypothalamic levels of various parameters in different groups of mice.

Treatment groups	MDA (n mol/mg protein)	GSSG/GSH	GP (m U/mg protein)	GR (m U/mg protein)
(1) Non-heated mice	6.9 ± 0.6	0.55 ± 0.16	318 ± 39	182 ± 18
(2) Heated mice treated with vehicle solution	12 ± 2^a^	2.24 ± 0.38^a^	87 ± 18^a^	76 ± 12^a^
(3) Heated mice treated with melatonin 0.2 mg/kg	11 ± 1^a^	2.42 ± 0.3^b^	76 ± 15	72 ± 11
(4) Heated mice treated with melatonin 1 mg/kg	6 ± 1^b^	0.98 ± 0.21^b^	185 ± 25^b^	144 ± 16^b^
(5) Heated mice treated with melatonin 5 mg/kg	4 ± 1^b^	0.42 ± 0.15^b^	369 ± 41^b^	192 ± 19^b^

Samples were measured 4 hours after whole body heating for non-heated groups. ^a^Compared with non-heated groups (*P* < 0.05); ^b^Compared with group 2 (*P* < 0.05). Data are means ± S.E.M. of 12 mice per group.

**Table 5:** Effect of heat exposure on serum levels of blood urea nitrogen (BUN), creatinine, alanine aminotransferase (ALT), aspartate aminotransferase (AST), and alkaline phosphate (AP) in different groups of mice.

Treatment groups	BUN (mmol/L)	Creatinine (mmol/L)	ALT (U/L)	AST (U/L)	AP (U/L)
(1) Non-heated mice	8 ± 1	27 ± 2	35 ± 4	111 ± 8	299 ± 23
(2) Heated mice treated with vehicle solution	23 ± 1^a^	75 ± 3^a^	129 ± 5^a^	508 ± 22^a^	533 ± 31^a^
(3) Heated mice treated with melatonin 0.2 mg/kg	21 ± 2	69 ± 4	130 ± 6	492 ± 21	505 ± 28
(4) Heated mice treated with melatonin 1 mg/kg	15 ± 2^b^	52 ± 2^b^	85 ± 3^b^	274 ± 16^b^	395 ± 19^b^
(5) Heated mice treated 5 mg/kg	10 ± 1^b^	43 ± 3^b^	67 ± 4^b^	188 ± 12^b^	364 ± 17^b^

Samples were measured 4 hours after whole body heating or the equivalent time period for non-heated groups. ^a^Compared with non-heated group (*P* < 0.05); ^b^Compared with group 2 (*P* < 0.05). Data are means ± S.E.M. of 12 mice per group.

**Table 6:** Effect of heat exposure on serum levels of adrenocorticotrophic-hormone (ACTH) and corticosterone in different groups of mice.

Treatment groups	ACTH (pg·mL^−1^)	Corticosterone (ng·mL^−1^)
(1) Non-heated mice	392 ± 98	142 ± 24
(2) Heated mice treated with vehicle solution	984 ± 127^a^	505 ± 26^a^
(3) Heated mice treated with melatonin 0.2 mg/kg	1001 ± 145	518 ± 28
(4) Heated mice treated with melatonin 1 mg/kg	1668 ± 162^b^	719 ± 31^b^
(5) Heated mice treated with melatonin 5 mg/kg	2167 ± 176^b^	854 ± 35^b^

Samples were measured 4 hours after whole body heating or the equivalent time period for non-heated groups. ^a^Compared with non-heated group (*P* < 0.05); ^b^Compared with group 2 (*P* < 0.05). Data are means ± S.E.M. of 12 mice per group.
